# Liquid–Liquid Phase Separation in Cardiovascular Diseases

**DOI:** 10.3390/cells11193040

**Published:** 2022-09-28

**Authors:** Yuanxi Mo, Yuliang Feng, Wei Huang, Ning Tan, Xinyi Li, Minwen Jie, Tong Feng, Hao Jiang, Lei Jiang

**Affiliations:** 1 Guangdong Provincial Key Laboratory of Coronary Heart Disease Prevention, Department of Cardiology, Guangdong Cardiovascular Institute, Guangdong Provincial People’s Hospital, Guangdong Academy of Medical Sciences, Guangzhou 510080, China; 2 Botnar Research Centre, Nuffield Department of Orthopaedics, Rheumatology and Musculoskeletal Sciences, University of Oxford Old Road, Headington, Oxford OX3 7LD, UK; 3 Department of Pathology and Laboratory Medicine, University of Cincinnati College of Medicine, Cincinnati, OH 45267, USA; 4 Laboratory for Aging and Cancer Research, National Clinical Research Center for Geriatrics, West China Hospital, Sichuan University, Chengdu 610041, China; 5 State Key Laboratory of Biocontrol, School of Life Sciences, Sun Yat-sen University, Guangzhou 510275, China; 6 Guangdong Provincial Geriatrics Institute, Guangdong Provincial People’s Hospital, Guangdong Academy of Medical Sciences, South China University of Technology, Guangzhou 510080, China

**Keywords:** liquid–liquid phase separation, cardiovascular disease, membrane-free organelle

## Abstract

Liquid–liquid phase separation (LLPS) is a biochemical process in cells that can drive proteins, RNA, and other molecules to concentrate into droplets. These droplets do not have a lipid membrane but rather exist as distinct organelles relative to the surrounding environment, and act as biochemical reaction chambers. In recent years, significant progress has been made in the study of LLPS, especially in the neurodegenerative disease, cancer, and virology fields, but little is known about LLPS in cardiovascular disease (CVD). In this review, we discuss the current understanding of the mechanism and biological functions of LLPS, particularly its roles in regulating CVD.

## 1. Introduction

Cardiovascular disease (CVD) is the leading cause of death worldwide. Prevention of CVDs has been a focus of interest. In the past 10 years, cardiovascular disease research has mainly focused on non-coding RNA [1]. However, the molecular mechanisms leading to CVDs are still not fully understood.

Liquid–liquid phase separation (LLPS), a process that facilitates protein–protein and protein–RNA interactions, has an important role in many diseases. However, the role of LLPS in the cardiovascular system has yet to be extensively studied. In this article, we review the potential connection between phase separation and CVD and provide new solutions for the prevention or treatment of CVD.

## 2. Characteristics of LLPS

The germ granules/P granules found in *Caenorhabditis elegans* (*C. elegans*) in 2009 were found to undergo LLPS, thus, introducing LLPS research [2]. Over the years, the LLPS characteristics have gradually been realized. Specifically, LLPS is a phenomenon underlying the formation of membrane-less organelles in the cytoplasm and nucleoplasm via weak multivalent interactions. The surrounding cytoplasm responds by forming a distinct membrane-free compartment. Identifying the characteristics of LLPS is essential for evaluating whether LLPS has occurred. In fact, the droplets formed by LLPS exhibit two physical characteristics. First, the droplets are spherical in isolation due to surface tension, and proteins that undergo phase separation show round droplets after in vitro purification [3]. In vivo, the spherical droplets appear as punctate aggregates after staining [4]. Second, the droplets undergo fusion, and several droplets can fuse to form a larger droplet. Tools used to identify LLPS include differential interference phase contrast microscopy and fluorescence recovery after photobleaching (FRAP) experiments [3]. Additionally, some scientists have found a way to observe phase separation in vivo. For example, local signal enhancement of P bodies was observed in the germ cells of *C. elegans*, indicating that the P bodies underwent local coagulation, similar to droplets observed under a microscope [2]. Subsequent studies have increasingly revealed that phase separation occurs in other prokaryotic and eukaryotic cells [5].

Under pathological conditions, various proteins can aggregate through LLPS, which hinders normal cellular function and accelerates the development of diseases. For example, TDP-43 and FUS were found to accumulate in the degenerative motor neurons of amyotrophic lateral sclerosis (ALS) patients through LLPS [6].

In recent years, studies have found that some specific particles are formed by LLPS. For example, ribonucleoprotein (RNP) particles [7], also known as RNA/protein or RNA particles, are crucial structures found in the cytoplasm and nuclei of living cells involved in a variety of diseases, including neurological, cardiovascular, and reproductive diseases and cancer, through their role in the processing and storage of RNA. Cellular RNPs are mainly divided into the following two types: nuclear granules, such as Cajal bodies and nucleoli, and cytoplasmic granules, such as stress granules (SGs) and P-bodies. Because RNPs are rich in RNA and proteins, RNA–protein and protein–protein interactions occur in these structures. Among RNPs, those that contain mRNA at their core are called messenger ribonucleoprotein (mRNPs), which can regulate the translation, localization, and conversion of mRNA. These mRNPs have been shown to be closely associated with neurodegenerative diseases. The persistence of mRNP complexes, such as SGs and P bodies, is related to neurodegenerative diseases, such as ALS, frontotemporal dementia (FTD), and frontotemporal lobar degeneration (FTLD) [8]. In the cardiovascular system, mRNPs can reach the sites of myofibril synthesis in cardiomyocytes through microtubule transport to mediate cardiac hypertrophy [9], which suggests that mRNPs participate in the formation of the membranous septum in the synthesis of myofibrils.

## 3. Regulation of LLPS

The assembly of membrane-less organelles by LLPS is a dynamic process that relies on the weak interaction between protein and protein or protein and RNA. This effect is often provided by specific regions between molecules, including the IDR region, hydrophobic amino acid region, charged region, etc. The assembly starts by forming an unstable core through weak interactions and then recruiting proteins to stabilize the core [10]. In neurons, this process relies on cytoskeletal proteins and molecular motors. It can transport and aggregate individual mRNPs/small mRNP particles into larger structures. That is to say, the entire RNPs are regarded as membrane-less organelles. Similar to membrane organelles, different organelles reach designated locations along microtubules and microfilaments through the action of actin, myosin, and ATP.

The LLPS is easily affected by many factors, such as light intensity, temperature, salt concentration, ATP concentration, post-translational modifications (PTMs), and chaperone proteins. In their study, Brangwynne et al. [11] developed a new optogenetics platform that regulates phase transition in living cells by controllable light; this platform induces phase separation by connecting the photosensitive protein PHR to the intrinsically disordered regions (IDRs) terminal by blue light irradiation. This method can help explore LLPS in cells. 

Temperature is involved in controlling phase separation [12,13], and an upper critical solution temperature (UCST) for LLPS has been identified. In vitro, protamine and protamine–multivalent ion complexes that had originally condensed were found to dissociate with increasing temperature, but when the temperature dropped, their aggregation resumed [14]. This shows that LLPS requires certain temperature conditions. 

Changing the concentrations of salt ions in solution is a widely studied method to induce LLPS. This is because DDX3X is more prone to phase separation in high-salt solutions, as salt ions change weak interactions between molecules, affecting the occurrence of phase separation. Moreover, ATP can affect the assembly of aggregates and act as a water-soluble growth aid to reduce the protein aggregation caused by LLPS [15]. Furthermore, some results suggest that various ATP-driven remodeling complexes regulate SGs [16]. First, ATP is the key driving force for phase separation. Moreover, ATP can maintain the protein concentration in the particles and, when proteins are present at a high concentration, ATP may counteract the tendency of IDRs to form amyloid fibers. 

Here, PTMs are the main mechanism regulating phase separation. Currently known PTMs affecting phase separation include methylation, phosphorylation, acetylation, and ubiquitin [17,18,19]. These PTMs promote or inhibit LLPS by changing disordered regions’ charge distributions and hydrophobic properties.

In vitro, the turbidity of a solution of acetylated DDX3X-IDR1 was lower than that of unacetylated IDR1 in the solution, indicating that the acetylation of DDX3X-IDR1 can weaken its LLPS [18]. For the same protein, different post-translational modifications will have different effects on LLPS. For example, a high level of tau acetylation is not conducive to LLPS and affects its interaction with microtubules [20], but phosphorylation of tau contributes to LLPS [21]. Interestingly, it has recently been discovered that ubiquitin is important for the regulation of LLPS. The ubiquitin-like protein ubiquilin 2 (UBQLN2) was found to regulate LLPS by directly regulating the fluidity of FUS–RNA complexes and kinetics of SG formation in the neurodegenerative diseases ALS and FTD [22]. Phosphorylation changes the frequency of LLPS by affecting the difference in charges between proteins, while ubiquitination can affect mRNA splicing. In most nuclear body proteins, ubiquitination can control splicing and promote the assembly of spliced aggregates through protein–protein interactions. 

**Figure 1:**
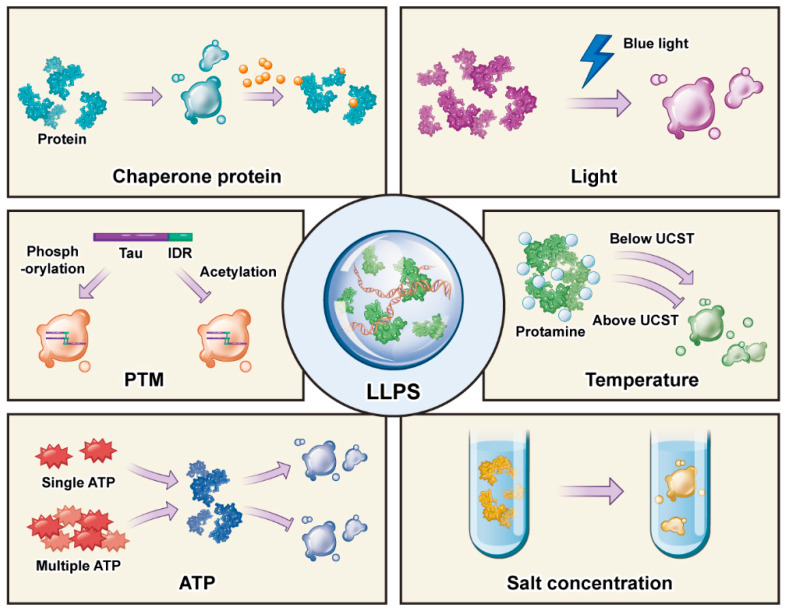
Factors that regulate liquid–liquid phase separation. Several factors, including light, temperature, salt concentration, ATP, PTM, and chaperones, can promote/inhibit liquid–liquid separation. Tau is an intrinsically unfolded protein. Abbreviations are as follows: PTM, post-translational modification; IDR, intrinsically disordered region; UCST, upper critical solution temperature.

Molecular chaperones are also important factors in regulating phase separation [23]. Chaperones affect phase separation mainly by inhibiting the formation of abnormal phase separation. High levels of the chaperone proteins HSP27 and heat shock protein 70 (HSP70) were found in SOD1-positive SGs, and HSP70 was able to prevent the formation of abnormal SGs [24]. This may be related to the fact that molecular chaperones bind to disordered regions of proteins, reducing the weak interaction between proteins. This protective mechanism of cellular evolution may prevent abnormal phase transition formation through the recruitment of chaperones (Figure 1).

## 4. Potential Role of LLPS in the Cardiovascular System

At present, exploring the biological function of LLPS is a critical task, and the exploration of its physiological function and its significance in diseases is required. Based on current evidence, phase separation is one of the mechanisms by which compartments form. Phase separation also mediates various biological functions, such as mitosis, growth, and development [25]. At present, phase separation is believed to cause the accumulation of proteins and diseases and to exert a protective role in organisms [26]. The main evidence for these functions is mostly from studies on neurodegenerative diseases. Most neurodegenerative proteins contain prion-like domains; although sufficient in vivo experimental evidence is lacking, these proteins, which have an important role in Alzheimer’s disease and ALS, may form LLPS in vitro. However, the biological function of LLPS in other systems has not been revealed. However, some evidence suggests that proteins may be capable of undergoing phase separation in CVDs. For example, Liao et al. used RBDmap to capture 1148 RBPs in cardiomyocytes and found 393 unique RBPs in cardiomyocytes [27] This indicates that, in CVDs, a considerable portion of proteins may undergo LLPS, because the interaction between proteins and RNA is an important form of phase separation. At present, two protein databases, LLPSDB and PhaSePro, contain data specific to LLPS; many proteins in these databases have been proven to undergo phase separation in vitro and have an important role in CVDs (Table 1) [28]. These databases provide the possibility to explore LLPS in the cardiovascular system. For example, HnRNPA1 is a key protein that regulates RNA metabolism in cytoplasmic RNP particles, and its *C*-terminal region is a key region for mediating dynamic LLPS. It may serve as a core scaffold to recruit miR-124, Drosha, and DGCR8 to coordinate the proliferation of vascular smooth muscle cells (VSMCs) and endocardium formation [29,30]. Moreover, BRD4 is a protein that phase-separates under high-salt conditions. It has been studied in cancer in the past. In recent years, its unique regulatory function has been found in heart failure and atherosclerosis. Additionally, BRD4 may have a regulatory role at the transcriptional level by recruiting transcription factors through phase separation. For example, the lack of BRD4 at the transcription level can cause the occurrence of dilated cardiomyopathy [31,32]. Furthermore, MED1 is also an important transcription factor for the heart. It has been able to form droplets on the super-enhancer. At the same time, it acts as a bridge between the cardiac enhancer and the promoter, directing the specific expression of more than 5000 genes. Mutations in MED1 can cause acute heart failure in the heart [33,34,35].

### 4.1. CVD and Intrinsically Disordered Proteins

Inherently disordered regions of proteins do not adopt a fixed tertiary structure, which contributes to the formation of multivalent interactions or misfolding in LLPS. Multivalent interactions are various non-covalent interactions formed when two molecules are combined. The IDRs of proteins are important factors driving phase separation. When proteins aggregate, an IDR undergoes a disorder-to-order transition [53] When all lysine residues in DDX3X-IDR1 were mutated to glutamine, a decrease in turbidity during LLPS in vitro was observed [18]. This showed that IDRs have an important role in phase separation.

Currently, there are more than 15,000 know proteins with an IDR. The IDRs can help proteins recognize and accelerate their interaction with other proteins and carry out alternative splicing, PTM, protein fusion, and insertion or deletion functions. Through database analysis of 487 proteins whose sequences were extracted from Swiss-Prot, 198 disordered regions among 101 proteins were predicted, and CVD-related proteins with disordered regions were very abundant [54]. Among these proteins, PDE4D may be involved in the occurrence of stroke through atherosclerosis. According to predictions, both the N-terminus and C-terminus of PDE4D contain disordered regions. The N-terminus participates in functions, such as phosphorylation and multimer formation, and the C-terminus is mainly related to dimer formation. Disordered regions can bind the SH3 domains of certain proteins, such as the Src family tyrosyl kinases lyn, fyn, and src. Furthermore, the SH3-binding domain of fyn kinase can bind proteins to induce LLPS [55]. Additionally, PDE4D may also interact with the SH3-binding domain of the kinase Fyn and induce phase separation.

### 4.2. LncRNAs-Associated Phase Separation in CVD

**Figure 2:**
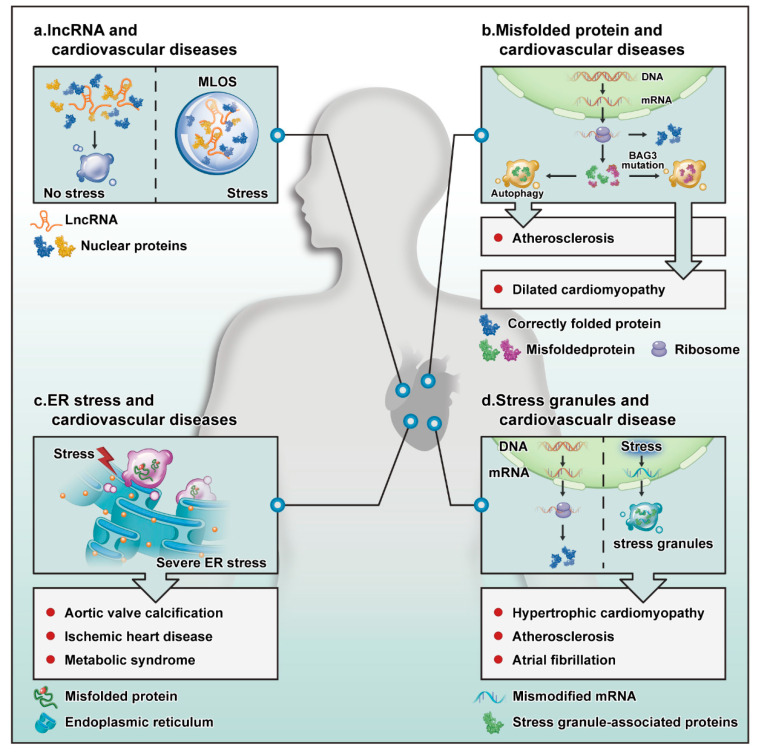
Potential role of LLPS in the cardiovascular system. (**a**) When stress occurs, lncRNAs become the core of droplets formed by liquid–liquid separation. (**b**) A correctly translated protein (blue) will perform its normal biological activities. Some misfolded proteins (green) are eliminated by autophagy to protect against atherosclerosis. Mutated or excessively produced proteins (red) accumulate, causing DCM. (**c**) The accumulation of a large number of misfolded proteins in the endoplasmic reticulum leads to endoplasmic reticulum stress, which can affect aortic valve calcification, ischemic heart disease, and metabolic syndrome. (**d**) Mistranscribed or modified mRNAs are eliminated by SG formation in the cytoplasm. This process can affect the progression of hypertrophic cardiomyopathy, atherosclerosis, and atrial fibrillation. Abbreviations are as follows: ER stress, endoplasmic reticulum stress; MLOs, membrane-free organelles.

As non-coding RNAs, lncRNAs regulate epigenetic, transcriptional, and translation processes in organisms. Interestingly, lncRNAs can form the core of the separated droplets (Figure 2a). Furthermore, SGs often use untranslated mRNA as a scaffold and nucleosomes in the nucleus cluster with lncRNAs or pre-mRNA scaffolds [56]. Architectural RNAs (ArcRNAs) have a key role in particle assembly. The currently known arcRNAs are primarily mRNAs, and only five lncRNAs can act as arcRNAs, as follows: (1) the cores of nucleosome paraspeckles contain NEAT1, which mainly inhibits apoptosis and induces antiviral genes during viral infection and pregnancy establishment [57]. (2) Amyloid (amyloid bodies) uses IGS as its core and function to repair proteins in the body and regulate ribosome formation. (3) Satellite III in nuclear SGs is mainly involved in the separation of RBPs and transcription factors. (4) A heat shock RNA (hsr-omega) in omega speckles in *Drosophila melanogaster* is involved in normal development [58]. (5) *Saccharomyces cerevisiae* meiRNA in the Mei2 site is involved in the process of meiosis [59]. At the same time, lncRNA can also promote the phase separation of other proteins and recruit more proteins into the droplets, which also shows that lncRNA has an important role in phase separation [60].

Many lncRNAs have been reported to be closely related to CVD [40,61], and these relationships are usually related to the sponge function of microRNAs. So far, these lncRNAs have not been reported to undergo phase separation in cardiovascular disease. These lncRNAs may also help the formation of phase separation cores in cardiovascular diseases and may act as auxiliary tools to promote the formation of other phase separations. Whether these lncRNAs can also be used as arcRNAs to regulate downstream genes is worth investigating. Mannen et al. and Chujo et al. explored two methods to identify new nuclear bodies, namely RNase sensitivity screening and transcriptome screening of semi-extractable RNA, respectively [62,63]. These two methods can identify lncRNAs to assist in stent formation in CVDs.

### 4.3. Protein Misfolding in Cardiomyopathy

Protein folding is an important step by which proteins adopt a three-dimensional tertiary structure. To ensure normal operation in the body, proteins must be correctly folded to carry out their normal biological roles. Misfolded proteins are eliminated through the autophagy system. When regulation of protein expression or degradation pathways fails and degradation pathways are destroyed, proteins accumulate, and pathological changes occur. Interestingly, proteins that are not eliminated due to failure of the autophagy system may cause proteasome disease through phase separation (Figure 2b). In FTD and ALS, proteins accumulate due to targeted damage to degradation pathway proteins, such as FUS and TDP-43 [64,65]. Proteins may incorrectly accumulate for the following reasons: (1) autophagy and protein quality control (PQC) disorder can cause incorrect protein accumulation. When PQC proteins mutate, misfolded proteins cannot be cleared and accumulate, resulting in phase separation. For example, when BAG3 is deleted, or a residue in the BAG domain is mutated from glutamate to lysine (E455K), the interaction between BAG3 and HSP70 is reduced, thus, decreasing the stability of the PQC system [66]. (2) Mutated proteins cannot be eliminated by autophagy. The RNPs can cause protein aggregation through LLPS. Pathological aggregation is often caused by structural misfolding due to protein mutation [67]. (3) The mislocalization of proteins is caused by protein mutations, e.g., the FUS protein accumulates in ALS because most missense point mutations in FUS are concentrated on fragments that encode the nuclear localization sequence at the C-terminus. These mutations affect the nuclear localization of FUS and cause FUS to aggregate in the cytoplasm [65].

Recent studies have shown a close relationship between dilated cardiomyopathy (DCM) and protein mutation. For example, homozygous disruption of the Bag3 gene causes the fulminant form of DCM, and mutated CRYAB, which encodes α B-crystallin, was detected in patients with DCM [68,69]. A recent study showed a phase separation phenomenon caused by abnormal RNP particle accumulation in DCM caused by RBM20 mutation [70]. This also revealed a certain association between protein mutation and phase separation. It is known that BAG3, a multidomain chaperone protein, is a component of the HSP70-BAG3 complex. In addition, as an important signal transduction node, BAG3 is part of the Hippo-2 signal transduction complex in the Hsp70–Bag3–LATS1 pathway, which regulates protein aggregation [71]. Interestingly, the mutation of BAG3 can cause BAG3 to accumulate and HSP70 to aggregate [72]. This aggregation is induced because BAG3–HSP70–HSPB, a key complex, acts as a retrograde transport in autophagy in normal cells to promote the isolation and removal of irreversibly misfolded proteins in the PQC system. The mutation of BAG3 destroys the complex and prevents cells from carrying out normal PQC, resulting in misfolded protein accumulation and protein-related diseases.

### 4.4. The Cardiovascular System and Endoplasmic Reticulum Stress

Endoplasmic reticulum stress refers to the destruction of endoplasmic reticulum homeostasis and the accumulation of unfolded or misfolded proteins in the endoplasmic reticulum, leading to dysfunction of the endoplasmic reticulum and affecting cell functions, such as cell cycle regulation [73]. Atherosclerosis and ischemic heart disease are common CVDs and the main causes of myocardial ischemia. Endoplasmic reticulum stress may be an important mechanism for the further aggravation of myocardial ischemia. A recent study showed that the inhibition of endoplasmic reticulum stress could reduce the hypertrophy of cardiomyocytes and cardiomyocyte damage [74].

Wang et al. demonstrated that aortic valve calcification caused by hypercholesterolemia is related to endoplasmic reticulum stress in animal experiments [75]. It has also been reported that endoplasmic reticulum stress can downregulate HDAC6 and promote aortic valve calcification [76], which may be because the endoplasmic reticulum is an important intracellular calcium reservoir in cells. When endoplasmic reticulum stress occurs, endoplasmic reticulum dysfunction leads to disordered calcium regulation and aortic valve calcification (Figure 2c).

In addition, endoplasmic reticulum stress is related to metabolism. A high-fat diet may cause excessive production of oxidative free radicals through high levels of low-density lipoproteins (LDLs), destroying the homeostasis of the endoplasmic reticulum and causing endoplasmic reticulum stress [77]. A high-sugar diet can also damage endothelial cell function through endoplasmic reticulum stress. All these results prove the close relationship between endoplasmic reticulum stress and CVD.

### 4.5. Atherosclerosis and Autophagy

Phase separation plays an important role in promoting the formation of autophagosomes and the process of autophagy (Figure 2b) [78]. Atherosclerotic heart disease is a CVD with a high incidence, and the accumulation of abnormal protein promotes the progression of atherosclerosis and heart failure; therefore, the removal of these organelles and proteins by autophagy exerts protective effects against atherosclerosis. Autophagy has dual effects on the progression of atherosclerosis. Excessive autophagy or a delay in the macrophage autophagy response will aggravate the occurrence of atherosclerosis [79]. The ATG family genes are autophagy-related genes that play an important role in vascular regulation. When the ATG5 gene was knocked out, atherosclerosis in mice was aggravated [80]. Recent studies have also found that this gene and other ATG family genes can regulate protein autophagy through phase separation [81]. Therefore, we speculate that autophagosomes are formed through phase separation, which recruits abnormal proteins to autophagosomes to achieve degradation, thereby affecting the process of atherosclerosis. In the future, exploring the role of phase separation in atherosclerosis will help delay the progression of the disease.

### 4.6. Atherosclerosis and SGs

Stress granules, particles that form in response to stress, are involved in regulating CVD (Figure 2d). For example, the accumulation of SGs can be observed in VSMCs and macrophages and affects the process of atherosclerosis [82]. Oxidized LDL (oxLDL) promotes the formation of such stress particles. As the first barrier in blood vessels, endothelial cells are most susceptible to stress caused by atherosclerotic stimulation, such as the modification of LDL levels and inducers of mitochondrial and oxidative stress, making it possible to explore the formation of pressure particles in endothelial cells.

Atrial fibrillation is the most common clinical arrhythmia and is also an important culprit of strokes caused by cardiovascular and cerebrovascular diseases. Enhanced cardiomyocyte activity is considered a strong stimulus for atrial fibrillation, and the heart may form SGs in response to such stimulation. The production of SGs was observed in primary cardiomyocytes and HL-1 cells after 1 h of pacing [83], which may be due to the excessive activity of cardiomyocytes that increases the oxidative stress of the myocardium and induces the production of SGs. 

Hypertrophic cardiomyopathy is a genetic mutation-related disease. Some of these proteins, such as CALR3, NEXN, and TPM1, are also contained in SGs, which indicates that these proteins may regulate hypertrophic cardiomyopathy through phase separation. These proteins may have a role in the disease process through phase separation. For example, NEXN, a key heart-specific Z disc protein, can protect the Z disc from mechanical trauma [84]. Recent research shows that mutations in this protein could cause hypertrophic cardiomyopathy in zebrafish, and mutations in the corresponding gene can cause DCM in humans. The NEXN protein contains two actin-binding domains (ABDs) and a coiled-coil (CC) domain, and *N*-terminal ABD and CC domain mutants have been observed. These mutants exhibit local accumulation in the cytoplasm, while the ABD fragment from wild-type NEXN accumulates in the nucleus [85]. Thus, the mutation of NEXN causes an error in its localization, similar to the effect of FUS mutation.

## 5. Discussion

**Figure 3:**
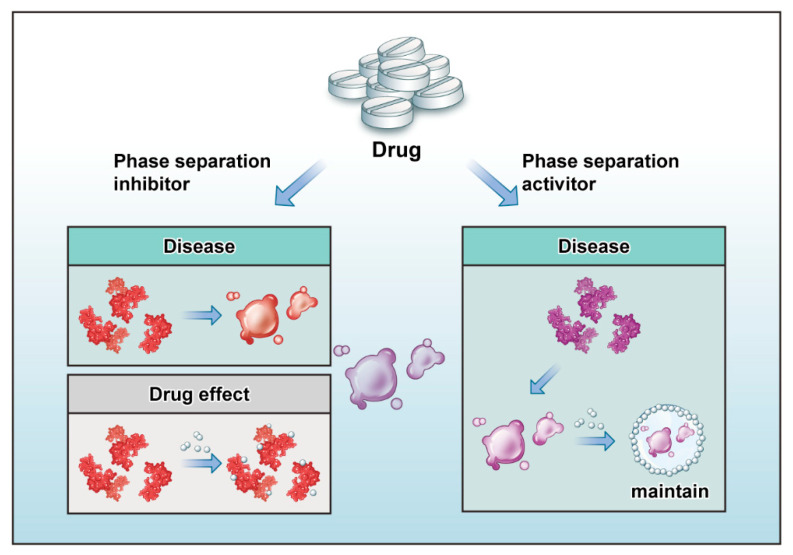
Possible ways through which drugs affect phase separation. Possible ways through which drugs promote/inhibit phase separation: (1) drugs may act as short peptide inhibitors of phase separation and inhibit phase separation through competitively binding to key sites of phase separation; (2) drugs can maintain phase separation by adjusting the external environment of phase separation, such as pH, salt concentration, etc.

Research interest in phase separation is growing, and phase separation is expected to become a new form of epigenetic regulation. In the past few years, related research has focused mainly on the biological mechanisms of phase separation, while few studies have revealed the function of phase separation. However, unlike the function of phase separation in the nervous system, cancer, and aging, its function in other systems is still unclear. Although no direct evidence has revealed phase separation in the cardiovascular system, current evidence suggests a connection between phase separation and CVD. For example, lncRNAs can act as arcRNAs to recruit proteins, affecting the progression of CVD. In addition, misfolded proteins may accumulate in cells due to mutations and cause diseases, such as DCM. Furthermore, the occurrence of autophagy affects the progression of atherosclerosis, and aortic valve calcification, ischemic cardiomyopathy, and metabolic syndrome are caused by network stress. Moreover, SGs can form in diseases, such as hypertrophic cardiomyopathy and atrial fibrillation. These studies provide a basis for exploring LLPS in CVDs. Additionally, phase separation may become a bridge to connect drugs and diseases. Phase separation is also an important link between drug production and drug transportation. Adding a water-soluble antagonist to a drug could be used to promote phase separation of the drug to maintain its stability [86]. Research has shown that phase separation has an important role in diseases and drug preparation. Phase-change materials may act as a medium for drugs in the future. Therefore, the design of related drugs from the perspective of phase change has great potential in the cardiovascular field. Herein, we highlight two factors related to the future of drug design (Figure 3). First, if protein phase separation exacerbates a disease, in the future, we could develop short peptides to inhibit phase separation by competitively binding sites of phase separation or other key proteins that are expressed during phase separation. This will inhibit phase separation and, thus, the effects of these proteins. Second, if protein phase separation has an important biological role in diseases, we can use drugs to adjust the external environment of droplets containing these drugs, such as the salt concentrations and pH, or adjust key proteins to achieve stable phase separation.

In basic research, the methods used to adjust phase separation still have limitations, which mainly include the following: (1) the mechanism of phase separation is still not fully understood; (2) the ability to interfere specifically with phase separation is difficult; (3) in vitro experiments still have technical limitations, and good in vivo experimental models are lacking. Therefore, the ability to study phase separation is limited, and much work remains to be carried out before drugs that transform phase separation are made. In the future, the following work should be performed: (1) to prove that the technology of phase separation is in need of a breakthrough; (2) the means of interfering phase separation are in need of a breakthrough, such as finding specific interfering phase separation drugs or protein mutants is important; (3) the application of phase separation in drug transformation is of great clinical significance. 

Despite its many limitations, phase separation has become a potentially important method of protein regulation and new regulatory mechanism in the field of proteomics. We believe these limitations can be resolved in the future and that phase separation can exert a major role in regulating CVDs.

## Figures and Tables

**Table 1:** Proteins that are phase-separated in the cardiovascular system.

Protein Name	Cellular Location	Database	Function
hnRNPA1	Nucleus	LLPSDB	Promotes the proliferation of VSMCs and the formation of new intima [29]. Acts as an important protein that maintains the development of human embryos whose mutations can cause congenital heart defects [30].
P63	Nucleus	LLPSDB	Regulates the development of embryonic stem cells in the heart [36]. Participates in the proliferation of smooth muscle cells regulated by microRNA [37].
hnRNPA2	Nucleus	LLPSDB	Promotes stem cell smooth muscle differentiation and embryonic arteriogenesis [38].
BRD4	Nucleus	LLPSDB	Accelerates the uptake of lipids in the blood vessel wall during the aging process and aggravates atherosclerosis [31]. Regulates endothelial-mesenchymal transition and cardiac fibrosis [32].
MED1	Nucleus	LLPSDB	Affects heart development and DCM [33]. Has an anti-atherosclerotic effect [34]. Prevents myocardial ischemia-reperfusion injury [35].
Numb	Cell membrane	LLPSDB	Maintains cardiac morphology and the differentiation of cardiac progenitor cells [39].
MEG3	Nucleus	LLPSDB	Participates in cardiac fibrosis and diastolic dysfunction [40]. Regulates angiogenesis [41].
FMR1	Nucleus	PhaSePro	Participates in myocardial cell injury during ischemia and reperfusion [42]. Protects cardiomyocytes from myocardial injury induced by lipopolysaccharide [43].
NONO	Nucleus	PhaSePro	Prevents the excessive proliferation of cardiac fibroblasts [44]. Promotes the instability of atherosclerotic plaque and increases the incidence of plaque destruction [45].
DAXX	Nucleus	PhaSePro	Inhibits the proliferation of vascular smooth muscle [46]. Mediates endothelial cell apoptosis [47]. Mediates myocardial apoptosis during stress or ischemia-reperfusion [48].
dyrk1a	Nucleus	PhaSePro	It may play a role in myocardial changes in Down syndrome [49]. Inhibits cardiomyocyte hypertrophy [50].
GATA3	Nucleus	PhaSePro	Mediates endothelial cell migration [51].
AGO2	Nucleus	PhaSePro	Mediates diabetic cardiomyopathy [52].
